# Science Diplomacy in Ecuador: Political Discourse and Practices Between 2007 and 2017

**DOI:** 10.3389/frma.2021.656969

**Published:** 2021-05-11

**Authors:** Kleinsy Bonilla, Milena Serafim, Efraín Bámaca-López

**Affiliations:** ^1^State University of Campinas, Campinas, Brazil; ^2^Institute for the Development of Higher Education in Guatemala INDESGUA, Guatemala City, Guatemala; ^3^Federal University of São Carlos, São Carlos, Brazil; ^4^Faculty of Agronomy, University San Carlos de Guatemala (USAC), Guatemala City, Guatemala

**Keywords:** science diplomacy, Ecuador, STI policy, STI capacity building, 2007–2017, Latin America, Science International Cooperation, sustainable development

## Abstract

The decade 2007–2017 was a period in which the Republic of Ecuador experienced a series of economic, social, cultural, and political transformations. This research focused on science, technology, and innovation (STI) changes with implications for Ecuador's foreign policy. One of the core components incorporated was Ecuador's engagement with foreign governments and various regional and global actors to further scientific and technological advances. These far-reaching collaborations aimed to reduce gaps the country experienced in science and research. Moreover, to incorporate Ecuador into worldwide initiatives to tackle cross-border issues, such as climate change and environmental sustainability. These measures included academic and scientific mobility through an extensive scholarship program, the *Prometeo* Fellowship Program, the Yasuni ITT Initiative, the creation of emblematic research universities, and certain guidelines released by the Ministry of Foreign Affairs and Human Mobility of Ecuador related to these policies. This article reports on qualitative research in which President Rafael Vicente Correa Delgado's political discourse was analyzed, along with key STI policies promoted in his administrations. The objective of this study was to establish different implications from the Science Diplomacy perspective (SD); particularly, reflecting on the consistency between the political rhetoric and the policy implementation. Evidence suggests that the political discourse materialized into concrete STI policies that could partially explain positive transformations in various aspects of the STI context in Ecuador. Institutional strengthening, international mobility (inward and outward), increased scientific output, and foreign policy practices involving SD which can be traced in the studied period. SD strategies could have been more effective and lasting if they were not discontinued upon Correa's departure from the Presidency.

## Introduction

The decade 2007–2017 was a period in which the Republic of Ecuador experienced a series of economic, social, cultural and political transformations. This research focused on the aspects related to science, technology and innovation (STI). Since Rafeal Correa's presidential campaign “knowledge revolution” fostering the international engagement of Ecuador STI was embedded in the political discourse (García, 2015; Aguirre, 2020). Emphasis was put on prospects of building a stronger domestic institutional setting and implementing learning processes from better international practices in STI. Various public policies addressing different areas of the STI context in Ecuador were prioritized, with an intended impact on the foreign policy. During the period 2011–2017, Ecuador's international relations incorporated purposeful guidelines to foster closer collaboration with countries of greater scientific and technological advancement to reduce the considerable science and existing gaps the country experienced.

Regional and South-South scientific cooperation were also placed at the center of Ecuador's foreign policy, diversifying the nature, characteristics, and origins of its partnerships. Also, transboundary issues such as climate change and environmental sustainability were included in various STI policies. Among these policies were: academic and scientific mobility through an extensive scholarship program promoted by the National Secretariat of Higher Education, Science, Technology, and Innovation (SENESCYT), the *Prometeo* Fellowship Program, the Yasuni ITT Initiative, Yachay City of Knowledge, and Yachay University of Technology, Ikiam Amazon University, and science diplomacy related guidelines issued by the Ministry of Foreign Affairs and Human Mobility. In this context, this research was guided by the following questions: **(a)** Which experiences of science diplomacy can be identified in the political discourse and the STI policies implemented in Ecuador during the 2007–2017 period? and **(b)** Which nuances are especially relevant to Ecuador from the science diplomacy perspective?

The main objective of this work was to analyze governmental strategies for Science Diplomacy (SD) in Ecuador from 2007 to 2007, seeking to highlight the advances and limitations related to institutional transformation, international mobility—in and out of the country, scientific output (journal publications, and patents), and foreign policy practices. Most similar studies present and analyze one or another public policy strategy developed during the period analyzed. The present work adds a more comprehensive look, verifying, through bibliographic review, documentary analysis, and in-depth interviews with participating actors—directly or indirectly. This was done by analyzing the political discourse at the highest level and examining relevant public policies promoted between the years 2007 and 2017. This paper is divided into four main parts: **(i)** presents the main arguments and overview on SD studies, **(ii)** research methodology, **(iii)** the main STI policies and practices of scientific diplomacy and international engagement during the Correa Government, and **(iv)** the analysis of conditions for implementation.

## Conceptual Framework

Diplomatic relations involving STI do not have a consensual term that defines them and is commonly used by actors who participate and promote international relations and cooperation in the area (Acuto and Kaltofen, 2018). Two denominations are the most common: Scientific Diplomacy (Varela et al., 2017) or Science Diplomacy (Arroz and Mendoça, 2016). Although there is no consensus around the definition of SD, the Royal Society and the American Association for the Advancement of Science (The Royal Society-AAAS., 2010) state that SD mainly seeks to (i) inform foreign policy objectives with scientific advice; (ii) facilitate international scientific cooperation, and; (iii) use scientific cooperation to improve international relations between countries and to solve shared problems. SD studies recognize the importance of STI in international relations and collaboration between countries. Economic globalization and contemporary challenges, such as pandemics, climate change, global security, sustainable development, are common phenomena with shared risks and impacts globally (Beck, 1992). SD is conceptualized by Acuto and Kaltofen (2018) as the practice whose objective is to maintain, cultivate and expand relations between countries. Therefore, the role of scientists and scientific information in world politics permeates the spectrum of global policy like never before (Acuto and Kaltofen, 2018). Given this, STI through scientific collaborations has become an increasingly important aspect of world politics and a critical element to ensure that the design of public policies and political decision-making take scientific evidence into account (Fernandez-Poluch, 2015).

Some countries have institutions whose purpose is to create a dialogue between researchers and political decision-makers, like the Council of Finish Academies and Italy's World Academy of Sciences (Council of Finish Academies, 2019). Other countries have used SD to strengthen innovation capacities (Flink and Schreiterer, 2010), promote capacity via exchange in the fields of higher education and STI (Mendonça, 2015) or bilateral agreements for the circulation of knowledge and technology transfer (Dolan, 2012), among others.

Latin America is gaining more space on the world stage, especially in cooperation with emerging countries, such as the BRICS; however, its peripheral condition of development, as ECLAC studies have shown for a long time (Prebisch, 1987; ECLAC., 2011), has not allowed the region to achieve a more symmetric relationship with developed countries. The efforts undertaken by SD in countries such as Ecuador require minimally consolidated scientific, technological, and innovative capabilities and the ability to approach advanced research and development centers in other countries. Otherwise, these efforts are being increasingly slowed down or losing radicality in their goals due to the lack of material conditions to propel them.

Matus (1993) points out that governing does not depend only on the government plan or project. Two other conditions must also be considered: governance and government capacity. The Correa Government had very promising SD proposals and strategies. There was initially a support base in the legislature and other state and non-state actors, but the third element is just as important as the other two: government capacity. This refers to the intellectual, organizational, and technical capital that the government team and other organizations/institutions—as the scientific community—need to have to leverage and implement the strategies. These capacities need to be built and consolidated before strategies, and public policies are developed and implemented. Without these capabilities, the trend is that in the short and medium-term, the results will be below expectations. This is a very common and shared phenomenon among Latin American countries. Political immediacy is antagonistic to perennial state policies.

## Methodology

The research data was collected by means of a qualitative methodology using three types of sources. First, to fulfill the study's general objective, bibliographic reviews and desk research were carried out. Second, to collect primary data, an instrument to conduct semi-structured interviews was designed and applied to key participants^1^. In order to elaborate the list of key participants, a strict set of criteria was established to include diversity and complementary/contrasting perspectives. The criteria were three-fold: (a) **Perspective**: Four complementary viewpoints: Foreign Policy, STI Policy, Scientific Community and International perspective. (b) **Deliverance ability**: middle to high-level decision-making position, and (c) **Relevance:** Significant exposure to the SD practices. The operationalization of the criteria is presented in Table 1. The potential participants involved those who: are/were public servants, officers, staff of public institutions, members of the scientific community, and/or directive members of an international (regional/global) organization. Also, those who had participated (or studied) directly or indirectly in the Correa Delgado Government's SD strategies. The themes addressed by the interviewees belong to the public domain and represent perspectives based on their own experiences. From a preliminary list of 35 potential interviewees, 25 responses were effectively collected. As part of the study, a search was made for guidelines issued by the Ministry of Foreign Affairs and Mobility, relevant to science, technology, and innovation toward the missions and members of Ecuador's foreign service. Special emphasis was placed on the Embassy of Ecuador in the Republic of Korea. This foreign mission hosted a pilot plan involving the appointment of an officer in charge of specific issues related to cooperation in education, science, technology, and innovation. That department was identified as Knowledge and Technology Transfer Analysis (MREMH., 2019). This position included duties and responsibilities focused on promoting science diplomacy initiatives in the bilateral relation Ecuador-Korea.

**Table 1:** Perspectives—balanced interviewees viewpoints and sector of action and experience.

**Perspective**	**Operationalization of the profile**	**No**.
Foreign policy perspective	Former/Current Diplomat, Officer/Staff of the Ministry of Foreign Affairs, and Human Mobility	8
Scientific community perspective	Scholar, Researcher, Member of the Scientific Community, with relevant understanding of STI in Ecuador	7
STI policy perspective	Political analysts, Connoisseur of STI in Ecuador, Former/Current Public Officer in any of the identified SD practices	7
International perspective	Experienced professional, current/former representative - high-level officer in STI international initiatives, a regional organization involving the participation of Ecuador in STI processes	3
Total		25

From the application of the semi-structured interviews, nearly 24 h of audio material was collected. Each interview session had an average duration of 45 min. Various platforms were used to conduct the interviews, including Google-meets, Zoom, and WhatsApp. The interviews were transcribed into text files, codified and analyzed to determine patterns, trends, common content, and contrasting views. In the discussion section of this article, block quotations are included to facilitate reading and elaborating on the main findings. Identities of participants are not revealed, instead the respective codes for each relevant participation are cited.

## Key STI Policies and the International Engagement of Ecuador

As a result of the inquiry, a group of flagship policies involving SD practices were identified. Table 2 summarizes the findings, and this section discusses their content, objectives, and general scope.

**Table 2:** Science diplomacy and key policy practices Ecuador 2007–2017.

**Policy**	**Description**	**Science Diplomacy Features**
Communication The Citizen Link	Permanent space prioritizing the communication of design, implementation, achievements, and challenges to emblematic STI policies	Constant interaction between stakeholders: public institutions, the Foreign Service, communication to the citizens, and engagement with the wider society
*Prometeo* Program	A comprehensive Fellowship program aimed at attracting accomplished and experienced scientists: foreigners and Ecuadorians residing overseas	To construct local research capacity and build international partnerships with their institutions of origin
Yachay Tech and Yachay City of Knowledge	The first planned city in Ecuador focused on the generation of a boosting STI Ecosystem with the creation of a world-class research university at the center	Planned and implemented based on successful experiences in knowledge cities, particularly Incheon Free Economic Zone and Silicon Valley, the policy included a very active set of foreign policy practices
Ikiam Amazonas University	An emblematic research university created as part of a biodiversity reserve in the Ecuadorian Amazons focused on global issues: climate change and environmental sustainability	Developed internationally benchmarked teaching, research, and community service mission envisioned as a living natural laboratory over an assertive practice of building global partnerships
Yasuni-ITT	Proposal to leave underground 846 million barrels of heavy crude oil found in the ITT fields, preventing the emissions of 407 million metric tons of carbon dioxide with co-responsibility by contributing half of the revenue coming from the international community	Transitioning from a fossil fuel-based economy toward a model of sustainable development, with widespread use of renewable energy, respect for biodiversity, and social equity with the involvement of foreign governments, multilateral organizations, responsible businesses, and citizens around the world
SENESCYT Scholarship Program	Nearly 20,000 scholarships benefiting outstanding Ecuadorian students for international mobility	Building the human power to establish a path for talented Ecuadorians to create a scientific (research) career learning from better international practices in higher education. The program involved active participation of the Ministry of Foreign Affairs, the international missions of Ecuador, and fostering cooperation between Ecuadorian and international higher education institutions with the support of SENESCYT
Embassy of Ecuador in Korea Appointment of the Knowledge and Technology Transfer Officer	The Ministry of Foreign Affairs and Human Mobility appointed a specific unit at the Embassy of Ecuador in the Republic of Korea aiming at promoting science and technology transfer within the scope of bilateral relations	STI as one of the pillars of the bilateral relations between the two countries, which influenced various areas such as investment (energy, infrastructure), preferential treatment in the promotion of several initiatives involving training, Official Development Assistance, cooperation

### The Political Discourse Addressing the International Engagement of Ecuador Science, Technology, and Innovation During the 2007–2017 Administration

President Rafael Correa Delgado's administration established a permanent and comprehensive system of communication that lasted during both of his administrations. This included three main components: regular meetings with representatives of the local and foreign press (*conversatorios*), mobile Cabinet meetings in the territories (*gabinete itinerante)*, and, at the core was the *Enlace Ciudadano* (Citizen Link). The *Enlace Ciudadano* was a radio and television program (also widely streamed on social media) in which information about major policies were aired to the country. The first program was transmitted on January 20th, 2007, and the last on May 20th, 2017, with 523 transmissions during the entire decade. The *Enlace Ciudadano* was the main channel to inform the direction, measures, and details of the Ecuadorian Government's policy priorities during this period. The program's format experienced several modifications; however, the inclusion of science and technology policies was a constant practice. In the first years, there was a segment called “Announcements about Science and Technology.” Various studies have highlighted how science, technology, and research were among the most recurrent topics in the program (Cañizares and Vanegas, 2012; Plua, 2014; Cerbino et al., 2017; Chavero et al., 2017). These citizen links were spaces for meeting the population; although the real benefits of such dynamics are questioned concerning what was invested in said spaces for dissemination and communication of government actions.

Frequent mentions about the international engagement of Ecuador in science and technology were also consistently present (Aguirre, 2020). Excerpts of a key Participant about the inclusion of STI international engagement in public interventions of President Rafael Correa Delgado:

No doubt about the importance that various science and technology policies had in the public interventions of President Rafael Correa. Yachay [City of Knowledge and University] was a recurrent topic in the Citizen Links, also the transformations promoted in the building of scientific and research infrastructure in the country. Yachay was also at the center of the foreign policy and the international agenda in the travels of President Rafael Correa Delgado. Conversely, Yachay Public Company had an entire team responsible for planning visits, receiving international delegations, promoting cooperation agreements, and providing academic tours. President [Correa Delgado] visited Stanford, Harvard, and other world-class universities to build partnerships and make numerous alliances. The support to Yachay Tech, Ikiam University, and the SENESCYT was evident at the highest political levels (Participant EC10).

Interviewees also provided statements about the specific attention that was given to science and research activities in the international relations of Ecuador.

President Rafael Correa Delgado had a permanent interest in promoting science, technology, and higher education cooperation in each of the international trips. He made sure to include in the official delegations accompanying the Presidential team, Congress people in the relevant Commissions to STI, top officers of SENESCYT, public universities, Yachay Public Company, and the Yasuni ITT Initiative during the years the policy was active. Also, the guidelines to prepare de agendas always included high-level meetings with authorities of the best universities in the countries of destinations, visits to world-class research institutions, and bilateral/multilateral encounters, when possible, to promote the emblematic universities, the transformation of the energy matrix and cooperation in different topics. For example, the promotion of closer ties in health cooperation with UNASUR [Union of the South American Nations] and research cooperation with various countries in Asia, particularly China and Korea (Participant EC11).

### The *Prometeo* Program

The *Prometeo* Program was an initiative of the Ecuadorian Government implemented between 2011 and 2017. This program's main objective was to “develop research capacities of higher education institutions and governmental entities to strengthen strategic sectors of Ecuador” (Echeverria and Diaz Flores, 2018: 12). It consisted of the mobility of accomplished and experienced researchers through fellowships to engage in scientific activities in the Ecuadorian territory. The main research areas were energy and innovation, life and natural resources, economics, business education, administration, social and behavioral sciences, and art and culture. A total of 848 *Prometeo* Fellows participated in the program with monthly stipends ranging between US$4,300 and US$6,000 (Celi, 2019), which raised questions about the amount allocated to such actions. As some interviewees expressed: the general idea of the program was perfect. What should be analyzed is whether the investments correspond to the benefits received in short, medium, and long term. A participant with relevant experience in the *Prometeo* Program pointed out:

A change was perceived in Ecuador, and that increase in investment had effects in [the process of] building scientific and research capacities in Ecuador. I think that the *Prometo* Program had great relevance to the government of Rafael Correa. Certainly, various shortcomings appeared during the implementation, and those failures attracted a lot of attention. For example, some fellows who claimed to have a doctorate degree were discovered in a lie and failed to prove such academic qualifications. These [shortcomings] happened because many changes were introduced very quickly. Hence the government did not have enough human power to implement all the new policies simultaneously, and the application and screening processes were unclear or simply involved incompetence of some mid-level officers (Participant EC2).

The *Prometeo* Program was widely advertised through the Foreign Missions of Ecuador accredited in different countries. Moreira-Mieles et al. (2020) and Van Hoof (2015) establish a relevant connection between the implementation of this policy and the significant increase in Ecuadorian scientific publications.

The program was initiated to re-incorporate Ecuadorian scientists or the scientific diaspora, but later expanded to bring scientists from other nationalities. Ecuador managed to attract nearly 1,000 scientists who were incorporated in universities and public research institutes. Experts, both Ecuadorians who were abroad and foreigners who met certain criteria, came to Ecuador and shared experiences to develop the STI system (Participant EC13).

The *Prometeo* Program was ended in 2017, reporting an estimated investment of 54 million US$ dollars (Celi, 2019) with the last Fellows completing their activities in 2019.

### Yachay—City of Knowledge and Yachay Tech

The new Constitution of Ecuador was approved in 2008. The National Development Plan *Buen Vivir* 2009–2013 provided a legal framework for President Rafael Correa (2007–2017) to promote a set of policies that sought to boost sustainable economic growth in a redistributive and inclusive manner. This included territorial inclusiveness and environmental sustainability. Two of these policies concentrated significant attention in this respect: Yachay—City of Knowledge and Yachay Technology University. According to official documents, Yachay's main objective was to become a city entirely dedicated to research, innovation, and production of various high-tech products and services (Yachay., 2012; Gómez-Urrego, 2019). The plan was to achieve this goal by bringing together public, private, and academic actors and institutions into one place. The realization of Yachay would enable the transition from an economy historically dependent on the extraction and export of commodities to an economy based on the generation of knowledge-intensive technologies and systemic innovation (Fernandez Gonzalez et al., 2018). The city project, located in the north of Ecuador, started in 2012 and should be completed in 2040 with the formation of the Yachay metropolitan area (Gómez-Urrego, 2019). The project, developed by the Korean firm IFEZ (Incheon Free Economic Zone), from the Republic of Korea was a big inspiration for Yachay. The guidelines contained in the project considered urban ecology, land use, mobility, urban dynamics, and civic integration (Ecuador, Plan Maestro, 2013).

The project sought to improve and create housing, commercial businesses, and communication networks that were not addressed by other strategic government projects. It also planned to promote *Buen Vivir* in rural areas and strengthen food sovereignty, as well as diversity and cultural heritage for sustainable development. At the core of Yachay City was the Yachay University of Technology. In 2014, Yachay Tech received its first student population, with professors from universities and technological institutes from several countries. Based on the nucleation of Yachay Tech University, and its relationship with research institutes, technology transfer centers, high-tech companies, and several public and private institutions, Yachay City would be the first technological park in Ecuador. Its main contribution to this grand-scale policy would be to structure an arrangement between the university, research, and industrial complexes, capable of developing innovative companies and fostering entrepreneurship.

At that time [2007–2017], the Yachay [City of Knowledge] was being prepared with this innovative approach. It was the first experience in Ecuador to have a city of knowledge, so it was impressive. A strong new institution was created: Yachay Public Company, to administer the entire long-term plan. The institution had a specific international cooperation team focused on fostering cooperation for higher education, science and technology (Participant EC10).

The city was planned to follow an industrial policy to promote technological development to export goods with high added value, which included an advanced strategy in the scope of international relations (Gómez-Urrego, 2019). Analyzing the trajectory of Yachay, Gómez-Urrego (2019) states that it was generated as a simple import or application of a foreign model (Fernandez Gonzalez et al., 2018), neglecting the trajectories of specific actors. This can be seen in the project's constant changes according to the political times and the actors involved.

### Ikiam Amazon University

Ikiam means Jungle in the Shuar indigenous language. Ikiam Amazon University was established on November 12, 2013, as one of the four “emblematic universities” created during the 2007–2017 administration. The institution was established with the specific Law (Ikiam, 2020, paragraph, 1–2) and started its academic activities in 2014. Since its conception, Ikiam Amazon University was envisioned as an institution to develop “internationally benchmarked teaching, research, and community service missions within the Ecuadorian Amazon (Wise and Carrazco, 2018).”

The University figures highlight that 62.75% of its research projects are internationally funded, denoting the importance of the intensive networking and international partnerships promoted by Ikiam Amazon University is the result of a series of policies aimed at strengthening the Ecuador higher education system, especially intertwined with the transformation of the energy and productive matrices of the country. The guiding principle of the university activities seeks environment sustainability and systemic competitiveness at the core of academic objectives. The implementation of this policy faced several challenges, such as not having laboratories and equipment at the beginning of their activities, which limited the institution's progress and highlighted the fact of having the largest natural laboratory in the world by its geographical location. Ikiam is an institution with coverage at the national level, but with particular emphasis on the region where it is installed—the Tena municipality in the Napa province. Wise and Carrazco (2018:342) maintain that this is a “case of top-down state-driven development model in which this university was established on principles of excellence, impact, and relevance.” An interviewee with relevant experience within Ikiam Amazon University indicated:

Ikiam is an example of internationalization. Since the early years of its structuring and organization stage, good practices from world-renowned universities were used as a benchmark. Three particular institutions provided guiding standards: Stanford University, Columbia University and Brazil's National Institute of Amazon Research INPA. Currently, it has signed 133 agreements with national and international counterparties. The scope and mission of Ikiam is different from that of Yachay Tech, that is why these two emblematic universities do not compete among them. While Yachay Tech was planned as a core component of a Planned City of Knowledge, Ikiam was envisioned as a natural reserve element. Ikiam promotes interaction of the academic community (students, professors, researchers) with high-level universities (Participant EC3).

From the science diplomacy perspective, the creation and implementation of Ikiam Amazon University is relevant considering the nature of the issues placed at the core of the institution's goals. Bacquet et al. (2018) reflect on the unpostponable environmental demands and pressures to generate science in a challenging socio-economic context such as the one present in a developing country like Ecuador. The reality built from the presence of Ikiam, opened possibilities of higher education to a distant population from the central cities of the country, but also generated spaces for discussion concerning the public administration of the invested resource; since the questioning for high salaries to certain advisers, did not stop generating disagreements among some sectors of the population.

### Yasuni-ITT

The Yasuní-ITT Initiative was another official project from 2007 to 2013, during the mandate of Rafael Correa. This initiative conditioned the maintenance of the Intangible Zone decreed in 1998 by the Jamil Mahuad government in a sector of the Yasuní National Park located between the Ishpingo, Tiputini, and Tambococha oil exploration quadrants. The intangible zone was decreed by the Mahuad government (1998–2000) with the purpose of not interfering in the territories of the native inhabitants located in the Amazon of Ecuador and keeping the biosphere reserve away from the oil exploitation that is carried out in various areas of the Ecuadorian Amazon rainforest. During the Correa Delgado government, it was proposed to leave part of the Amazon intangible zone unexploited conditioned to a compensation mechanism for the income not received. By not exploiting oil resources and keeping crude oil underground to the carbon market, the compensation would be made by the international community to the Ecuadorian State under the criteria of ecological economics, environmental economics, and natural resource economics. The Yasuní-ITT Initiative had five goals:

(1) effective conservation and avoidance of deforestation in the protected areas of Ecuador and other remaining ecosystems; (2) reforestation, afforestation, natural regeneration, and appropriate management of one million hectares of forest owned by small and medium landowners in lands that are currently threatened by degradation; (3) a change in the national energy matrix that increases the renewable energy generation and the national energy efficiency through energy savings; (4) social development in the areas of influence for the Yasuní-ITT Initiative, with programs for education, training, technical assistance, and the generation of productive employment in sustainable activities such as ecotourism and agroforestry; and (5) research and development in science, technology and innovation for the generation of goods and services based on bio-knowledge; and in addition, to implement the integrated watershed management (Vallejo et al., 2015: 176).

To develop the initiative, Ecuador designed foreign policy guidelines to promote co-responsibility of developed countries that are the largest global polluters, and with this, the Ecuadorian State would commit to leaving underground, indefinitely, around 856 million barrels of oil in the Yasuní ecological reserve, to avoid the emission into the atmosphere, of 407 million metric tons of carbon dioxide (which would be produced by the burning of these fossil fuels). In exchange, financial compensation was expected from the international community for a fraction of the estimated value for 50% of the profits received if these resources were to be exploited (about 350 million dollars a year). The funds raised by this operation would be reinvested in Ecuador in three lines: Management of 19 protected areas, a national reforestation program, and the energy matrix transformation. External contributions would go to a trust fund administered by the United Nations Development Program and, through an international body, the monitoring and implementation of the objectives necessary to make the transition to a new development strategy would take place (United Nations Development Programme (UNDP)., 2010). As compensation committed by some countries intended to impose conditions, of which most of the funds would be managed by a trust, several discrepancies arose between the potential contributors and the Ecuadorian government, for which reason the initiative was halted. The Yasuni ITT failed to be completed; nevertheless, it allowed for broadening the dialogue on power asymmetries and global governance.

### SENESCYT and the Mobility Scholarship Program

Based on article 26 of the Republic of Ecuador's Constitution, education is established as a right, and it is a responsibility of the State. At the same time, it is classified as “[.] a priority area of public policy and state investment, a guarantee of equality and social inclusion and an essential condition for good living [.]” (National Constituent Assembly., 2008). In this sense, the Organic Law of Higher Education in its article 182 (National Assembly., 2010), establishes that “[.] the National Secretariat of Higher Education, Science, Technology and Innovation (SENESCYT) is the body that exercise leadership in the public policies of higher education and coordinate actions between the Executive Branch and the institutions of the Higher Education System [.],” this became the legal foundation for the implementation of the public policy by the SENESCYT for the promotion of human talent in higher education. It is worth mentioning that through Executive Decree 555 dated January 9, 2015, the Institute for the Promotion of Human Talent was created, which came to replace the Ecuadorian Institute of Educational Loans and Scholarships (IECE), which had been created in 1971. The scholarship recipients became those “[.] contemporary heroes who go to study and learn in other places to return to their homeland and build a better nation for everyone [.]” (Speech by President Rafael Correa Delgado in the award of SENESCYT Scholarships 2012).

The primary purpose of this mobility of scholarship holders was to form and accumulate the human power needed for the development of Ecuador. It was compulsory for the awardees to complete their studies and return to Ecuadorian territory since human talent was considered necessary for the generation of wealth. All these actions were based on the National Development Plan *Buen Vivir*, of which its main axes guided the change of the national productive and energy matrices.

An attempt was made to respond to a historical debt that appeared with strong demands for higher education from marginalized minorities and who today disputed democratization at this level. But perhaps, a quota policy thus conceived and implemented responded better to the needs and demands of globalization, the new knowledge economy and new links between education and the economy—or within the Ecuadorian reality—to the needs of the Plan of National Development than to the subjects, their communities and histories and more real and deeper intercultural senses (Di Caudo, 2015: 210).

International relations were important in the mobility of Ecuadorians abroad. There was access to education of excellence in the best universities in the world. From the government's vision, its contribution was to induce many people to advance in their university education and seek improvements in the Ecuadorian territory upon their return. Investing on a “knowledge revolution” that would transform the productive matrix of the entire territory, going “from being a country of finite resources to generate infinite resources, because the cognitive matrix is fundamental for the change in the productive matrix” (official speeches cited in Di Caudo, 2015: 216). Among the main competences of the Under secretariat for Strengthening Knowledge and Scholarships, was the strategic management of public policy that supported and strengthened the training of excellence in all branches of knowledge, both in the facilitation of scholarships and the *Prometeo* program.

The public policy linked to professional and academic training, intended to contribute especially to those who otherwise could not have continued their training at another level. However, this distribution by quotas came with cultural shock to the beneficiaries since, on many occasions, this mobility was also accompanied by emotional loneliness.

This pilot quota plan designed and developed from public policy and implemented in a group of institutions to include young people ended up excluding them as direct beneficiaries. The meritocratic logic emphasized talent, formal education, competence, excellence instead of existing differences and alterities such as social class, ethnicity, sex, and disability (Di Caudo, 2015: 216).

With an estimated public investment of more than US$ 500 million (SENESCYT., 2012), the 2007–2017 administration implemented this extensive scholarship policy training Ecuadorians at the postgraduate level in the best universities worldwide. Such implementation required an intense dialogue with universities, academics, and international study centers, involving particular attention from the diplomatic missions of Ecuador on its growing scientific diaspora abroad. The passage of time helped to see favorable results, including situations that could be worked on in a better way, to be able to offer better management of resources, greater transparency, and better distribution of such benefits. At the time of conducting this research, the investment for such items has been seriously affected, to the extreme of being almost nil.

SENESCYT focused on supporting education of Ecuadorians in master's and doctoral programs overseas. Over 20,000 scholarships were awarded in total. There was also a program of insertion of these graduates to be absorbed in Ecuadorian universities upon return. These strategies shorten the time for the evolution of our scientific output. Ecuador jumped from <300 publications a year in a particular database to publishing 2,000 in the same platform (Participant EC13).

The central objective of the program was favorable, and it allowed the incorporation of a highly trained group. However, the question is that upon return, the country was not prepared in its institutions to achieve full placement of the talents trained. There was a deficiency in that regard. Deficiency that is still perceived since many of the people trained failed to join the promised job.

### Diplomacy for Science, the case of the Embassy of Ecuador in Korea

The Embassy of Ecuador in Korea represents a case of diplomacy for science in view of the particular guidelines and measures taken by the Ecuador Ministry of Foreign Affairs and Human Mobility to support various initiatives within the bilateral relation. The Embassy devoted particular efforts through the regular functions of career diplomats, in addition to the appointment of an officer in charge of issues specifically related to cooperation in higher education, science, technology, and innovation, and that had performance indicators relevant to the practice of science diplomacy between Ecuador and Korea. The model of rapid socio-economic development experienced in the Republic of Korea was a recurrent reference in the political discourse of Rafael Correa Delgado, particularly with respect to the role that research and development and the strong STI policies played in industrialization of this country (El Universo., 2012). In 2010, a State visit was organized by President Rafael Correa Delgado to follow a very comprehensive agenda dominated science and technology cooperation (Embassy of Korea in Ecuador., 2020). As a result of the preparations, the execution, and the development of the several areas of cooperation in STI reached during this visit, various processes unfolded.

The Korean model has been considered one of the main inspirations behind Yachay City of Knowledge, a major industrialization policy “*Refineria del Pacifico*.”

In September 2010, Rafael Correa Delgado embarked on a state visit to Korea in search for alliances, investment, and international cooperation in a variety of topics ranging from renewable energy to investment in the *Refiner*í*a del Pac*í*fico* (oil refinery to be built in the coast of Ecuador). The team behind the reconceptualization of the project of Yachay managed to include in the president's itinerary a visit to KIST (Korea Institute of Science and Technology) 44 and Incheon, which were, in their minds, materializations of the ideas they were developing. During the trip to Korea he [President Rafael Correa Delgado] visited several cities, including Songdo, and a variety of industrial complexes, to negotiate cooperation between the two nations in various topics, mainly around energetic investment from Korea in Ecuador. Correa Delgado became convinced that this was the kind of project his government needed: a project which combined both the educative transformation his government had promised and the change in the productive matrix, which had become the guiding sociotechnical imagined within the government (Gómez-Urrego, 2020: 130).

Interviewees also referred to the significance of the Korea-Ecuador bilateral relation for STI initiatives:

To my knowledge, creating a position specifically in charge of attending issues of bilateral cooperation in science and technology only occurred in the Embassy of Ecuador in The Republic of Korea. The initiative was innovative and meaningful. One of the elements that justified the position was the deep and close involvement of various Korean entities, including public institutions and private firms, in various critical STI policies. For example, the Incheon Free Economic Zone IFEZ, Daedeok Innopolis, from the city of Daejeon, were heavily involved in the planning of Yachay City of Knowledge, and SK energy had a key role in projects aiming at energy development in Ecuador. A particular emphasis was placed on students and researcher's mobility between the two countries. I remember there were Korean fellows under the *Prometeo* Program as well (Participant EC14).

The STI initiatives promoted in collaboration between the two countries received attention from Ecuadorian institutions in their territory. They had closed coordination with the Staff of the Mission of Ecuador in Korea.

An entire division of SENESCYT and YACHAY Public Company were devoted to attend the numerous common projects between Ecuador and Korea. The number of graduate students mobilizing to pursue master's and doctoral degrees in Korean universities grew exponentially during 2012–2105. Also, the number of delegations from public officers, trainees, business people, entrepreneurs going and coming between the two countries was significant in the same period. Everything needed important efforts to keep in check results, otherwise, it was hard to keep track of all the activities that were taking place. Unfortunately, the intensity and diversity of the cooperation diminished as the economic and political global context shifted since 2016. In addition, priorities on behalf of the Ecuadorian government change with the new administration after 2017, showing that STI cooperation does not occupy a high position in terms of priorities anymore (Participant EC6).

## Discussion and Findings

### Consistency Between the Political Discourse and Practice

A first analytic category is related to the consistency between the political discourse and the materialization of government proposals and actions. Ideally, a democratically elected government presents a Government Plan, which will, over time, be materialized in strategies and actions that allow the achievement of the pre-established objectives. However, for several reasons (i.e., political, material, financial or legal), what is observed is no linearity between proposal and action. There is some disruption between these two moments or contexts.

Excerpts from the interviews show this process and explain the difficulties and reasons that prevented, to some extent, the practice from accompanying the speech and political proposal. The material conditions and circumstances encountered made it difficult for the planned actions to be carried out satisfactorily to the fullest extent.

I think there was consistency between speech and action in the first years of President Correa's administration. First, they laid the foundations that would allow them to carry out actions. Then, they took concrete actions to improve science and technology sectors; however, as time progressed, it became evident that the discourse was amplified. I mean. the discourse was greater than actions. There were many plans, programs, and policies proposed, but the implementation and, therefore, achieving results fell short due to multiple factors. There were documents and planning instruments, and the problem was that there were not enough conditions for them to succeed. The sudden changes, raising the standards introducing, performance indicators in STI were disruptive and interpreted as burdensome. For example, the search for external sources of income for universities to support research was encouraged, but the system made the execution of external funds very difficult. The levels of bureaucracy and red-tape processes were enormous. On paper, everything seemed viable, but in practice, it was difficult to implement (Participant EC1).

In the excerpt above, it is possible to verify that insufficient financial resources and the excess bureaucracy were two aspects highlighted by the interviewee and that, to some extent, are among the factors of difficulty in implementation. Another widely commented topic by the interviewees was an overly optimistic speech made concerning the SD practice. The organization of face-to-face visits, the composition of its delegation, and the construction of the official agenda, for example, were carefully designed.

Probably the presidential speech was exaggerated, but it should also be mentioned that there was an undeniable stress placed in STI areas of the policy in his administration. I believe that the level within the country in relation to science and technology rose significantly. Professors were required to have completed graduate studies, it may sound obvious, but indeed, the universities were forced to invest in research and personnel, to raise their standards (Participant EC4).

Using the concept of Herrera (1975), which spells out discussions related to the implicit and explicit policy in STI, it is evident in our analysis of SD—from the interviews—that the Correa Delgado Government had an alignment between its implicit and explicit policy.

I think that the political discourse was consistent with the policy practice to a significant extent. The Ministry of Higher Education, Science, and Technology creation which previously did not exist was important. The newly established institutional setting allowed the universities to find themselves in need to make progress in their standards, advance faster and improve their quality. A university evaluation system was proposed, and also funds and other forms of support were generated at the public policy level. Ecuadorian students had access to mobility alternatives. There were funds for research to bring samples for analysis and engage in joint research projects. However, not everything was positive. The expectations were too ambitious for our daring realities. So, the problem with exaggerating the discourse is that you generate expectations that are not real, and I think that played against the very same process in the long run (Participant EC2).

Although the Correa Government had decision makers' desire and prioritized SD strategies and practices, which are extremely important elements to undertake a new diplomacy logic, it is clear that they are not enough. Barriers and difficulties were imposed at different times, such as financial resources, personnel, and university capacity. We will see some of these limitations and/or challenges in the next section.

### Underlying Transformation in the Science Diplomacy Practices of Ecuador 2007–2017

All the interviewees agreed that since the return to democracy in Ecuador in 1978 until 2006, the international engagement of Ecuador in science and technology was absent from the political discourse and the government initiatives. This is supported by literature (Herrera-García, 2016; Herrera-García et al., 2019), which explain how during several administrations, the prevalence of extreme neoliberal policies in all aspects (economy, health, education, etc.) resulted in the elimination of nearly all competencies in science, technology and innovation policies from the public sector, except modest measures taken by the administration of President Jaime Roldós (1979–1981) and Rodrigo Borja (1989–1992) which were focused on domestic agenda (Campaña, 2020). It was until the period 2007–2017 when the administration of Rafael Correa Delgado incorporated science and technology policies at the center of its discourse and practice (Salazar Diaz, 2016). Various interviewees referred to the importance of establishing modern responsive regulations to facilitate the engagement of Ecuadorian stakeholders in the STI in scientific activities, both in the domestic and the international spheres. These transformations went beyond the executive branch of the government. The establishment of a responsible legal framework for science diplomacy required the decisive involvement of the legislative branch.

The new Constitution of 2008 favored STI policies to a great extent. It was necessary to recognize the importance of such policies for the development of Ecuador and update our legal framework foundations in the country. The Government National Plan *Buen Vivir*, which created an institutional and legal framework, allowed all these processes to occur with established planning. It was an obligation for the allocation of public funding, for example, all proposals involving research were articulated to the pillars and objectives of the National Plan (Participant EC1).
Between 2007 and 2017, there was a strong impulse of international relations in higher education, science and technology, and innovation. In the context, especially the field of higher education, this first stage referred to the recognition of qualifications and degrees obtained in foreign institutions. At a later stage, we began to look for many relationships linked more to the cooperation of funds for access to scholarships because Ecuador had a strong scholarship policy during the period 2011–2016, in which significant resources were invested (Participant EC13).

A portion of the nuances of Ecuador's legal framework is related to the recognition of ancestral knowledge and traditional practices of wisdom, which is generally disregarded in western scientific practices (de Sousa Santos, 2010).

The constitution of 2008 represented a turnaround in the legal framework of Ecuador. It expressly incorporated several features corresponding to STI. This was, perhaps, the first Constitutions in the history of Ecuador that textually includes the right to benefit from scientific progress and its applications, which consists of national human rights. It also created the system for Ecuador to participate in international science and technology. The system comprises the institutions of higher education, plus research institutes that articulate science and technology with traditional [indigenous] knowledge. The ancestral inhabitants of Ecuador generated knowledge that has always been hierarchically subordinated and many times not recognized. Context such as the Ecuadorian science and technology landscape required articulating locally generated traditional knowledge with internationally accepted scientific metrics and standards. The entire higher education system was also shaken to its core. In 2010, the organic specific law for higher education was approved to introduce compulsory research activities that had been completely abandoned in Ecuadorian universities. The SENESCYT created a whole set of incentives, programs, funds, and mechanisms. The institution began the allocation of funds through competitive calls. The legal framework also created several public research institutes (Participant EC13).

Another consensus among interviewees was the importance of institutional building, after or at least in parallel to establishing an STI suitable legal framework. Before 2007, some institutions were created. However, the entire STI system had limited participation of the public sector, and the budget allocated to these sectors was minimal (Herrera-García, 2016; Herrera-Garcia, 2018).

The Science and Technology Council in Ecuador followed a different logic than that observed in neighboring countries such Brazil, Chile or Argentina. In Ecuador its creation was a consequence of the prevalence of the neoliberal paradigm with minimum state involvement in socio-economic issues. The Council obeyed the neoliberal stage of development, yielding poor results, limited only to some sectors such as agriculture. But in all the other fields, it was totally disconnected from the country's needs, without access to financial resources or key policies, so an organ was created that did not generate results. Evidence of that was creating an organ in charge of science and technology, yet the legal figure was a private foundation FUNDACYT that resulted from a multilateral loan from the Inter-American Development Bank. The Washington Consensus embraced by Ecuador did not consider STI as sectors for the participation of the State. Before 2007 Ecuador did not have research infrastructure in the country; one of the main issues or weaknesses that the university system had was the education level of professors. Few of them had postgraduate degrees and doctoral training, so the human capacities to conduct research did not exist. FUNDACYT failed to articulate the higher education system with broader development goals. That is why the creation of SENESCYT in 2007 with way stronger mandates, size, structure, resources, and the highest support of power reflected positive results for the STI Ecuadorian system (Participant EC13).

Interviewees also identified different moments in the transformation of STI sectors in Ecuador even considering the 2007–2017 decade in which this study is focused. Participant EC20 proposed three key moments:

Regarding the 2007–2017 STI policies, I see a clear differentiation in three stages: First, 2007–2010 the institutional building that introduced deep transformations requiring close coordination between the legislative and the executive branches of the government. Various laws were issued, institutions created, policies designed and the human power at different levels were appointed. and the foundations for the coming phases were laid down. This included an integral intervention and reorganization of the higher education system. A second moment 2010–2014 in which further policy implementation was sought with an identifiable assertiveness in science and technology. And a third stage, 2015–2017 in which the innovation component was further incorporated although the government experienced a decrease in public support and pressures by external factors (changes in the international prices of commodities and a major earthquake) shifted priorities in the public agendas (Participant EC20).

### Conditions for Effective Implementation of Science Diplomacy Practices in Ecuador 2007–2017

#### Building a Baseline of STI Capacities

Understanding the baseline in terms of STI capacities in low and middle-income countries from the southern hemisphere is an important departure point to engage in international STI processes. In the case of Ecuador, as some participants indicated, the scientific and research capacities were minimal before 2007. Moreover, the political discourse and the public agenda did not include these areas as part of the national priorities.

Our scientific capacities in Ecuador before 2007 were deficient, even for regional standards in South America. There were very few opportunities to develop research in all aspects, including human and institutional capacities. Local universities offered few opportunities for graduate studies. The opportunities came from external scholarships and international cooperation. If someone intended to pursue academic specialization, people had to look for international funding. The only other option was self-financing or applying for educational loans. There were no international scholarly exchanges as such. We were merely recipients of certain assistance and external collaborations, donations in a very vertical dynamic of international cooperation (Participant EC4).

Context matters, and it also involves the dynamics affecting the international engagement of countries with the scientific development characteristics of Ecuador. Similar conditions are shared by other nations in Latin America. In this sense, minding the numerous gaps in different aspects is critical for effective SD practices.

The language and timing of bureaucracy, politics, and diplomacy, differing from that of science, is another issue that scientific diplomacy seeks to reconcile in terms of how these two worlds move in a way that advances. For example, some international scientists from the *Promoteo* Program arrived in Ecuador with expectations to work intensively and put in place all their valuable capabilities, but they found in a very arid context. Many of them possibly started activities without equipment, reagents, laboratories, and minimal conditions to start activities. There were no human resources available, and there was not all this context to facilitate high-level scientific research. Frequently, universities and scholars in Ecuador don't understand why you have to present the results of an investigation at a conference. Therefore, they don't provide authorization nor financial support, which created obstacles to make progress. How can such capabilities be built? Ecuador had 10 years of significant effort as a country and many resources directed to science and technology with valuable but limited results. Certainly, the science level improved, with constant pressure to publish more articles, in Correa's time. After the end of his presidency, fewer universities continue to have that momentum (Participant EC9).
One of the biggest contributions to STI from President Correa Delgado was achieving social awareness of the importance of these sectors for the development of the country. Before his administrations such topics were absent from the public agenda. Thanks to various of its policies stakeholders were created with interests and involvement in science and technology (Participant EC20).

#### Constant Interactions Between Stakeholders From Different Sectors

SD policy and practice assume as a condition of success, a permanent interaction between stakeholders (Lorenzo et al., 2020), which is critical in a context such as Ecuador. Developing countries tend to have fragmented STI systems in which the scientific community, political actors, and private firms follow separate agendas and attend differentiated and opposed interests. In these conditions, foreign policy finds limited room for action. This is why the integral approach observed in the policy guidelines of Ecuador's international engagement in the 2007–2017 period provides an interesting case of coordinated efforts. Certainly, the challenges experienced in the aligning of diverting interest of multiple stakeholders were also mentioned.

Beyond the will of the authorities, international collaboration required active participation of professors, researchers, and administrative staff, on behalf of universities and research institutes. We needed to reach further using the usual approach: signing a series of agreements, with no follow-up and measure of results. The *Prometeo* Program sought in its first phase, to attract people with high profiles to Ecuador by giving them sufficient funds. It allowed them to interact with local researchers and other professionals to create scientific networks. The number of scientific publications increased, networks were also created to link other researchers at the national and international level. In various cases, the Fellows were also placed in private firms (Participant EC3).
There was a comprehensive national plan to change the productive matrix of Ecuador. The 2007–2017 administration aimed at Ecuador ceasing to be a country that is exclusively a producer of raw materials and commodities. Instead, turning to industrialization policies, diversify its production and export more sophisticated and added value goods and services. A critical component was the transformation of the energy matrix; therefore, significant resources were invested in building hydroelectric plants. Before 2007 Ecuador had to import electrical energy, as the country relied mostly on fossil fuels. It was even common to experience power shortages despite having possible sources of electrical energy. Just as the government was invested in hydroelectric power, it should have been an immediate action to train specialized technicians to operate the power plants. We need to be strategic with different sectors: energy, hydrocarbons, higher education, and science. Other sectors, such as the production sector, began to talk about training. There were dialogue tables that were super interesting, in which the famous coordination of strategic sectors was present (Participant EC4).

Various participants indicated that an aspect that needed more attention to assess the effectiveness of the SD practices during the Rafael Correa Delgado administration was the involvement of the private sector. The improvements in the interactions between public organization, political actors and civil society in STI related policy is recognized in general, however, the involvement of companies and private capital seem to have fallen short.

A significant shortcoming in the various S&T policies during the administrations of Rafael Correa in Ecuador's government was the limited involvement of the private sector. One of the explanations of this limitation was the high centralization of the initiatives in the executive branch of government. Even universities were directed which left little room for companies and private firms to engage (Participant EC16).

#### Diversification in the Building of Partnerships

A key feature of Ecuador's foreign policy during 2007–2017 was the diversification in the building of partnerships. Before 2007, the international agenda was concentrated in relations with powerful countries in the North, particularly the United States and Central Europe. However, during Rafael Correa Delgado's administration, STI international engagement, although also involved initiatives with traditional partners, expanded and diversified the partner countries. In particular, seeking closer relations with countries in Asia: China, Korea, Russia, Turkey, and Latin America: Argentina, Cuba, Brazil.

Between 2007 and 2017, Ecuador was an attractive partner with many opportunities to generate international STI collaborations. European countries like Italy, France, and Spain, were aware that Ecuador changed its mission and was growing well, and therefore many people had intentions to support that growth. Ecuadorian people were in many scientific international institutions. They could be found in the United States, United Kingdom, France, Italy, and Spain. The Ecuadorian community's perception overseas began to change from the previously known migrant work in domestic services to science-related careers (Participant EC2).
I think the country's positioning in its foreign policy strengthened, especially in the Latin American region. South-South cooperation on these issues was emphasized (Participant EC4).
Ecuador invested significant resources in developing its science and technology sectors, but we also needed that cooperation to share the investment pressure with the international community. Hence, much of the foreign relations in this context involved the idea of seeking financing from cooperation agencies, universities, or partner governments in general to finance scholarships. The focus was placed on strengthening ties with those states and organizations that were the priorities. The trend or the political program of Correa Delgado's government was to achieve a South–South STI cooperation perhaps. Ecuador became a drive in the creation of the Union of the South American Nations Council of Science and Technology [UNASUR]. The mandate was to extend the agenda that we had been promoted to expand at a regional level. This was done in terms of international relations and integration. Certainly, relations with the northern countries were also pursued, especially in Europe. It was considered a milestone for Ecuador to collaborate with one of the most competitive research centers in the world, located in Switzerland. SENESCYT supported that engagement process. It involved several contacts, visit trips, it was a permanent component of the agenda. Finally, it was possible to sign an agreement with the European Council for Nuclear Research (CERN). That enabled several Ecuadorian students and scholars to join different activities of the organization. In addition, direct cooperation was achieved between CERN and the National Polytechnic School of Ecuador, which resulted in the establishment of two research centers in Ecuadorian territory. SENESCYT worked closely with diplomats from foreign embassies accredited in Ecuador, and also with the Ecuadorian missions located abroad (Participant EC13).
I believe that positioning Ecuador's interests in STI with the involvement of foreign policy was more productive as we managed to diversify our partners. We had offices in Eastern European countries that were willing to collaborate with us. Hungary offered scholarships for co-financed Ecuadorians, which alleviated the heavy investment needed for opening more educational options overseas. Ecuador also benefited from scholarships and other STI cooperation from Portugal, China, and Russia. This also gave us a lot of support, with the USA continuing our collaboration but became less dependent. With the European bloc there were more defined schemes, and negotiating equality was more complicated. The complexity of regulations and standards were considerable. Nevertheless, Ecuador in those years [2007–2017] experienced intense transformations (Participant EC6).

### Challenges for Science Diplomacy in Ecuador 2007-2017

#### Instability in the STI Policy

Developing countries such as Ecuador share features of instability in their political and socio-economic processes that inevitably have a detrimental impact. Various interviewees indicated that, from their different perspectives, progress in the design and implementation of well-structured STI policies was undeniable during the 2007–2017 administration. However, sudden changes in the policy trajectories were a persistent characteristic easily observed in the successive governments of Ecuador since 1979, which remain present to a different extent in the administration of President Rafael Correa Delgado. Herrera-Garcia and Franco-Crespo (2019:27) sustain that “The STI policies [during 2007–2010] were unstable since there were several short-term policy initiatives, and that the implementation was far from the ambitious rhetoric and the great objectives set, limiting themselves to two instruments: postgraduate scholarships abroad and financing of research projects and development.” Some interviewees referred to instability in the STI policies, as we can see in the excerpts below:

I think you have to divide the STI transformation in Ecuador into two periods, 2007–2014 was one period, and from 2016 onwards, things changed, of which the momentum was maintained for a while but then it declined in the last 2 years. I am very pessimistic, unfortunately, reality makes me so. The processes that took place to improve the STI context were nearly reversed, university accreditation and devaluation systems were erased. Regulations about standards and quality protocols were mandatory, then in 2017, changes were introduced from the new administration, and now they are voluntary. There were modifications to the regulations, and the change of government was an opportunity for those who resisted change to return to the starting point (Participant EC2).

Herrera-Garcia and Franco-Crespo (2019:27) sustain that “The STI policies [during 2007–2010] were unstable, since there were several short-term policy initiatives, and the implementation was far from the ambitious rhetoric and the great objectives set, limiting themselves to two instruments: postgraduate scholarships abroad and financing of research projects and development.” Admittedly, despite the challenging socio-economic and political conditions that do not allow the continuity and expansion of STI policies, which are essential to support science diplomacy, it is verified that there was an effort in the Correa Delgado Government. This was dismantled from 2018 with The Organic Law of Higher Education. This panorama can be corroborated by the EC4 Participant's comment below:

The National Plan had indicators and guidelines with which all actors had to be aligned with. From being a nation with weak knowledge production history, Ecuador aimed at becoming a benchmark experience in the Latin American region. The country actually made progress if we compare the STI context in Ecuador before 2007 and after 2017. Regrettably, a backward heading path has been taken by the current administration (Participant EC4).

Unfortunately, this instability scenario in the continuity and improvement of public policies is not peculiar to Ecuador. It is a very common phenomenon in the Latin American context, which has countries with low democratic maturity in their societies, with social inequalities that are quite aggravated, thus also apparently reducing the urgency in investing in STI policies.

The centrality that STI policies had in the National Development Plan and the vehemence that the President observed when addressing them in his discourse had a double edge: gathering public support and making those policies a target of attack from political adversaries. The long-term nature of the intended transformation presented a complex context, making the initiatives vulnerable during transition of power and political instability. Grand endeavors such as Yachay City of Knowledge required planning, the fulfillment of different phases and stages, but the pressures to produce short-term results and the political rush due to high expectations generated clashes, tensions, and disputes, which brought a toll into the project (Participant EC10).
The science diplomacy practices in Ecuador still find it difficult to transform episodic and circumstantial interventions into systematic and integral policies. The period we are discussing [2007-2017] involved stability and sustained efforts, however once the leadership of President Rafael Correa was replaced, the initiatives were halted (Participant EC21).
Transformations in the science and technology context of a country takes time. The multiple pressures from urgent problems affecting societies such as Ecuador frequently divert attention and resources from those sectors, which ultimately affect the speed and depth of the changes. From an international perspective we observed the sound policies and well-intended plans promoted by the 2007–2017 administrations, but also recognized antagonistic forces and the inertia of the development process introducing ups and downs to the pace of progress in the international engagement of Ecuador in S&T (Participant EC23).

#### Mismatch in STI Capacities in International Engagement of Ecuador

Various policies such as Ikiam University and Yachay Tech were part of a broader set of capacity-building strategies that were aligned with international STI capacities. The ambition of these projects and other STI actions, policies, and strategies marked the Correa Government. The perspective that the social and economic development of a country derived from scientific and technological development boosted measures that would encourage the constitution of a robust research and development system that is adequate to the country's development.

As the Science Without Borders program in Brazil, Ecuador adopted a policy of training and qualifying human resources within the scope of Universities. The financing of research and development projects for the training of graduate students (master/doctor) abroad placed Ecuador on the international stage. The qualification of a scientific community is one of the most important pillars of building a country's STI capacities. However, it is necessary to recognize that the establishment of an internationalized scientific community and a set of elements, which support a country's scientific and technological capabilities, require steady actions and constancy in government measures to promote science, technology, and innovation.

As we can see in the excerpts below, the government's effort is recognized, yet it is also pointed out there was a lack of better understanding of how universities work and how equipment and technological infrastructure are important for the constitution of research and development infrastructure.

I think they are long-term effects; both are different models. Ikiam is embedded in a nature reserve, which is the largest natural laboratory in the world, in a way, it is a correct approximation. Yet, the project neglected the acquisition of technological equipment. I think there were many management errors among other mistaken decisions, a gap between the conception in its operation. I think there was a lack of knowledge of how universities work, they said that in <10 years it would be among the 100 best in the world, which is impossible; that shows ignorance of the academic system, of how research works. I believe that people involved thought it was possible [.].Yachay was a project for a technological society that was going to be a development pole for the country. For a project this grand and ambitious to succeed key questions needed to be asked. For example, all public research institutes in the country were going to be located in Yachay, an area of mountains. The planners were also putting the institutes that do oceanography, research on oceanic marine culture in the same infrastructure; it was illogical (Participant EC1).

Another widely commented aspect also refers to the time required for the maturation of the scientific bases necessary to leverage a national system of science, technology, and innovation. Participants EC2 and EC9 point out—below—these challenges and possible obstacles.

As a country at the stage of forming the scientific bases necessary to, at some point be able to flourish, sustained efforts are required. This is not something that overnight, then this support for training scholars was important at that time and it lasted about, 5, 6 years. It helped strengthening the universities. From abroad, I could see how the universities in my country were improving (Participant EC2).
The truth is, the gap is huge. I believe that we should have governments with more vision in this regard. Some people have no idea what science and technology means. I believe that the Correa Delgado government did yield results. Science awareness improved, and the administration brought and generated technology in the country. Then this idea of raising the quality of the universities, he created a body that monitors and supervises the quality of the universities, that body continues to function, I don't know how efficient it is, but it continues to work (Participant EC9).

It is clear that the “impetus of the Correa Delgado Government” (Participant EC9) brought a new perspective to the scientific community and to universities. The experience of these actors, in spite of any obstacles experienced and the retrogression of these measures in later governments, marks a new era for scientific and technological complexity.

#### Dealing With Resistance to Change

It is also necessary to address some aspects of resistance to the transformations brought about by the internationalized STI measures proposed by the Correa Delgado Government. Some university actors questioned these measures and even encouraged a certain resistance to the proposed changes, especially with regard to the Universities' quality assessment and measurement systems. Comments on the motivations of this resistance can be seen in the following excerpts:

There is a group that I think never agreed with outsiders coming to Ecuador in the scientific sphere for multiple reasons. They saw their status threatened somehow. They had gotten used to the fact that you already have an appointment and were never worried about working harder because nobody could replace them. Having outsiders entering the scenario meant they also had to improve their performance, and then it disrupted a status of immobility that universities had, some even began to talk about “scientific colonialism,” people who come with other ideas to impose processes on us. At this point, there was a change, there was also rejection from the people here, possibly because they did not understand much about the importance of this change demanded by competitive practices in academia worldwide (Participant EC2).

Without wishing to strain the discussion of the extent to which these mobility processes and the formation of the Ecuadorian scientific community are constituted by scientific colonialism, it is worth pointing out that this discussion is already recognized. Herrera (1975), one of the founders of the Bariloche Movement, already pointed out this process as a trend in Latin American countries for emulation of ST and economic development in central countries. The consequence of the emulation of scientific agendas is the atypicality and anomaly of the STI policy, which barely meets national challenges (Dagnino, 2016; Spatti et al., 2021).

I think that from the technical perspective, the policies were correct. Clearly, there is a cultural context where they settle and where there is and maybe more complexity, so you look at the policies and see the results, and they are policies that put Ecuador in a better position. Change frequently causes certain dissatisfactions. On the one hand, there was resistance within the institutions to the arrival of these foreigners because they put the efforts into question in individual and group terms what those university communities have done. I think there was also a lack of cultural understanding. I think we can say that the difficulties existed, but the results were positive. In the end, many Ecuadorian academics ended up learning a lot from the people who came from abroad (Participant EC13).

Another mapped resistance was that related to the reorganization and restructuring of the institutional settings. Participant EC18 elaborated in the local perception of “re-foundation approach” taken during the early years of the 2007–2017 administrations, not only in the STI areas, but also to the wider national spectrum. Fields such as public health and vaccine production attempted to shift from the “pharmacological *maquila* approach to build a R&D-based production ecosystem, but the local vested interests boycott the process.” Similar mentions were done in regards to the agriculture, energy, and mining sectors.

#### Science Diplomacy Human Power (Staff, Foreign Service)

The last analytical category refers to the strong engagement of human resources in Science Diplomacy missions and actions. Most of the interviewees recognized that, in the analyzed period, the strong strategy coming directly from the Presidency and the immediate high-level collaborators managed to set policy and measures into practice. As for the Ministry of Foreign Affairs, mix appraisal was shared by interviewees who remarked some positive aspects while others highlight difficulties encountered. Participant EC3 pointed out, there was an engagement by the President and his team to foster international cooperation.

The pursuit of science, technology and higher education collaborations permeated the international relations of Ecuador between 2007 and 2017. The President was the one who always led the composition of the agendas. The economic, political, and diplomatic agendas incorporated science, technology, and innovation components. In that sense, very important agreements were signed at the international level. I don't have the exact figure, but many countries with which alliances and cooperation were created (Participant EC3).

The creation of a diplomacy school, together with important changes in the choice of representatives of the Ministry of Foreign Affairs and representatives in diplomatic missions abroad, were measures that demonstrate the seriousness with which the Government dealt with diplomacy. In the excerpt below, the participant highlights these measures and his experience in actions that involved political and scientific engagement in solving national problems.

An attempt was made to transform the Ministry of Foreign Affairs regarding who the country's representatives were, or at least how these representatives were selected. A school of diplomacy was created to facilitate broader access to sectors of the society. Traditionally, Ecuadorian diplomats were members of elite families, specific affirmative actions were implemented to increase the participation of women, indigenous population and people from unprivileged backgrounds. These changes were positive, but consumed the first years in office with newly appointed diplomats taking time to understand the dynamics of international engagement in S&T. As a member of the Ecuador scientific diaspora I found it difficult addressing topics such as ocean acidification and the involvement of Ecuador scholars and diplomats, in part explained by ignorance but also lack of interest at the scientific level (Participant EC2).

In addition, there was an effort to engage public research institutes and universities in science diplomacy discussions and in scientific and technological cooperation projects. In this sense, institutional building became a recurrent issue during the studied period.

In 2007, the Ecuadorian Agency for International Cooperation was created, and it became dependent on the National Planning and Development Secretariat precisely with the vision that the issue of international cooperation, in general, had to respond to the objectives of national development. It was still an instrument for international relations but under the logic of integrated planning. The main objective was directing every international cooperation effort to strengthen local capacities. Within this logic, the creation and development of local capacities was already seen with a larger vision (Participant EC10).

The commitment for international cooperation actions to promote higher education, science, and technology was explicit. The most emblematic project was Yachay Tech, a city of knowledge, which became one the flagship policies of the Correa Delgado Government. Participant EC10 understands that an impulse was given that had never been given, since the implementation of a university evaluation system, funding for training human resources for the production of science and technology, until the implementation of coordinated work with embassies and the consulates to promote the Yachay project as an unprecedented scientific-technological park in the Latin American scope.

Some participants offered a critical perspective of the challenges that SD practices encountered within the Ministry of Foreign Affairs and Human Mobility in terms of restrictive attitudes in the exercise of diplomacy and called attention to the importance of expanding the understanding of diplomacy.

The Ecuadorian institutional context in which diplomacy is exercised is very particular. Worldwide, it is accepted that career diplomats might have stronger incentives to perform efficiently based on stability and dedication to building their career path. Meanwhile temporary appointed foreign service members could obey to conjuncture pressures rather than consistent institutional guidelines. This has not been the case of Ecuador, in which the prevalence of a conventional diplomatic practice has been restrictive leaving little room for a modern attitude in diplomacy. In other words, we continue limiting our diplomacy to trade and commerce, culture and official development assistance, without reaching beyond these interests (Participant EC19).
In the diplomacy promoted by Ecuador, we find a predominance of a restrictive perspective to the content of the Vienna Convention with focus in the promotion of economic (commercial diplomacy) and cultural (cultural diplomacy) interest of the country. Narrow view which has not accompanied the evolution of other aspects of diplomacy such as international scientific collaboration and engaging in global issues (Participant EC21).

## Conclusions

The explicit terminology “science diplomacy” might not be found in the political discourse and the policies implemented in Ecuador during the 2007–2017 decade; however, the analysis of the rhetoric and policies of this administration offer clear evidence of the emphasis placed on the science international engagement of the country. This period marked a clear turning point in Ecuador's recent history when significant transformations in the science, technology, and innovation contexts were intended from the highest level of decision making.

Evidence suggests various flagship policies encountered in the interface between science and foreign policy. Also, some of these policies aimed at fostering Ecuador's participation in tackling issues within regional and global reach. The constant exploration in the intersection of domestic STI interests and the international relations of Ecuador had different intensity and depth levels depending on the scope and dynamics of interactions between the stakeholders. Nonetheless, the highest levels of political leadership (President, Vice-president, and Ministerial level) permanently incorporated signs of support for SD.

Building STI domestic capacities proved to be a necessary condition for the science international engagement of Ecuador, and at the same time, the nuances of this case confirmed that SD practices have plenty of opportunities to be directed precisely to such purpose in developing countries: the construction of a stronger STI baseline. SD strategies could have been more effective if Ecuador had more consolidated scientific, technological and innovative capacities and, of course, an STI Policy more structured. In Latin American countries, discontinuities between governmental administrations do not allow cumulative movements to be constituted. Another important element is about government capacities. Most Latin American Countries need to strengthen technical bureaucracies to achieve cognitive and administrative capacities in light of the ambition and boldness of government projects. In any case, even without the use of “science diplomacy” as an explicit conceptual framework, the intersection between STI policy and foreign policy is palpable during the 2007–2017 administration in Ecuador.

The pursued science diplomacy practices in Ecuador during 2007–2017 can partially explain positive transformations and measurable improvements in various science and technology indicators, including but not limited to: institutional strengthening, international mobility (inward and outward) scientific production (publications, patents), and foreign policy practices involving science. Further research is recommended to further the discussions of SD studies and perspectives from the global south realities. Findings in this study indicate that, while the political discourse in STI was decisive, and financial resources were purposefully channeled to the identified SD policy practices, a series of challenges present in the Ecuadorian context diminished the extent of effective implementation. Consequently, results and impact were also curtailed.

## Data Availability Statement

The raw data supporting the conclusions of this article will be made available by the authors, without undue reservation.

## Ethics Statement

Ethical review and approval was not required for the study on human participants in accordance with the local legislation and institutional requirements. The patients/participants provided their written informed consent to participate in this study.

## Author Contributions

All authors listed have made a substantial, direct and intellectual contribution to the work, and approved it for publication.

## Conflict of Interest

The authors declare that the research was conducted in the absence of any commercial or financial relationships that could be construed as a potential conflict of interest.

## Footnotes

^1^This research was not submitted to the ethics committee because it involves interviews dealing with matters in the public domain, in accordance with Resolution No. 510, of April 7, 2016, in which it has the rules on the National Commission of Research (CONEP) non-mandatory submission and evaluation, as well as the interviewees will not be identified in the dissemination of the results, being attributed the codes and title of Participant.
